# Probing Residual Water in G‑Quadruplex Structures through Molecular Vibrations

**DOI:** 10.1021/acs.jpcb.5c04949

**Published:** 2025-09-19

**Authors:** Valeria Libera, Sara Catalini, Francesca Ripanti, Luca Bertini, Martina Alunni Cardinali, Francesco D’Amico, Andrea Orecchini, Caterina Petrillo, Marco Paolantoni, Alessandro Paciaroni, Lucia Comez

**Affiliations:** † Dipartimento di Fisica e Geologia, 9309 Università di Perugia, Via Pascoli, 06123 Perugia, Italy; ‡ Department of Chemistry, University of Basel, St. Johanns-Ring 19, CH-4056 Basel, Switzerland; § European Laboratory for Non-Linear Spectroscopy, Via Nello Carrara 1, 50019 Sesto Fiorentino, Florence, Italy; ∥ Department of Life and Environmental Sciences, 9294 Polytechnic University of Marche, Via Brecce Bianche, 60131 Ancona, Italy; ⊥ Dipartimento di Chimica, Biologia e Biotecnologie, Università di Perugia, Via Elce di Sotto 8, 06123 Perugia, Italy; # Elettra-Sincrotrone Trieste, Strada Statale 14-km 163,5 in AREA Science Park, 34149 Basovizza, Trieste, Italy; ∇ Istituto Officina dei Materiali (IOM)—CNR, Via Pascoli, 06123 Perugia, Italy

## Abstract

G-quadruplexes (GQs), noncanonical DNA structures involved in gene regulation and genome stability, exhibit a high structural polymorphism that is strongly modulated by their solvation environment. Here we employ a dual-spectroscopy approach, combining ultraviolet resonance Raman (UVRR) scattering with circular dichroism (CD), to study GQ dilute solutions during thermal unfolding. UVRR scattering enhances the vibrational features of GQs, providing a method previously applied to other biosystems but novel for GQs in probing solute–solvent interactions. By analyzing the O–H stretching vibrational band, which reflects the hydrogen-bonded water network, we reveal how water molecules interact differently with two distinct GQ conformers, namely, hybrid and parallel. We demonstrate that coupling UVRR scattering with CD spectroscopy allows for the correlation of vibrational properties with secondary structural features of the solute, even in spectral regions dominated by solvent contributions. Expanding this approach to other GQs may offer deeper insights into the critical role of solvation in GQ stability and function.

## Graphical abstract

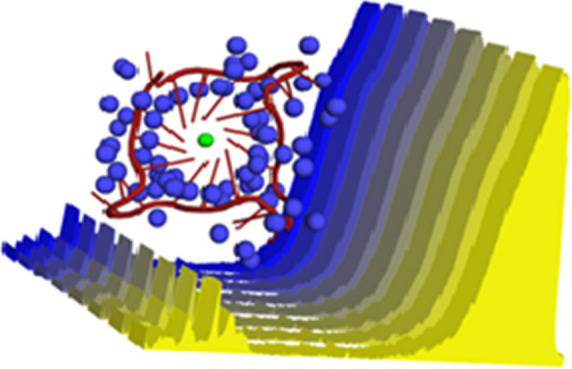

## Introduction

Guanine-rich nucleic acid sequences can fold into noncanonical, four-stranded secondary structures known as G-quadruplexes (GQs). GQs play crucial roles in key genomic functions such as transcription, replication, epigenetic regulation, and genome stability, with notable links to cancer biology.^1,2^ These discoveries have prompted extensive research into GQ functional mechanisms and their potential for therapeutic applications. GQs are composed of stacked G-tetrads, four guanine bases held together by Hoogsteen hydrogen bonds, with phosphodiester backbones creating grooves or cavities.^3^ Monovalent cations like Na^+^ and K^+^ facilitate GQ formation, making physiological buffers favorable for their assembly. GQ structures exhibit a high degree of polymorphism, being able to adopt different topologies depending on strand orientation, sequence length, and loop composition. The different conformations can be systematically categorized based on the glycosidic bond angle of the intervening bases, which adopt either anti or syn configurations, leading to parallel, antiparallel, or hybrid strand orientations.^4,5^ The assembly of GQs is driven primarily by noncovalent interactions between individual nucleosides and their surrounding environment, including hydration water molecules in the proximity of the biomolecule. These vicinal waters can reside in the grooves of GQs,^6^ whose size varies according to their topology: in parallel quadruplexes, all grooves are of medium width, whereas hybrid and antiparallel structures can exhibit three types of grooves: wide, medium, and narrow. Therefore, a distinct water network arrangement may be expected for each quadruplex conformation.^6^

Investigating these aspects is crucial because interactions with the solvent significantly influence folding, stability, and functionality of biomolecules. The hydration properties of various GQ topologies have been studied under crowded conditions^7–10^ and in water associated with crystal structures.^6^ However, there is limited literature addressing dilute solutions.^11,12^ Working at low concentrations (e.g micromolar regime) helps to prevent aggregation and uncontrolled topology changes.^13^ To explore this point, we employed a particularly effective technique, namely ultraviolet resonance Raman (UVRR) spectroscopy, which exploits the coupling of UV excitation wavelengths with resonant electronic transitions of DNA.^14,15^ Compared to the conventional Raman technique, this approach offers both chromophore selectivity and an up to 8-fold increased sensitivity for the label-free identification of a chromophore moiety in the molecule, even at biologically relevant micromolar concentrations. The Raman signal is amplified by several orders of magnitude, enabling the simultaneous investigation of the vibrational markers of both GQ and solvent.^12^ This capability allows for the integration of information from Raman experiments with data obtained from other techniques particularly suited for highly diluted systems. One such technique is circular dichroism (CD), which serves as an excellent tool for the rapid assessment of the secondary structure and folding properties of biomolecules.^16,17^ UVRR also enables the identification of solute-induced perturbations in water stretching vibrations, which vary in intensity and strength depending on the GQ topology.^12^ Additionally, it has been recently demonstrated that the OH stretching of water experiences a preresonance signal enhancement, particularly effective in amplifying higher-frequency contributions related to more disordered water configurations.^18^ The UVRR-CD dual technique presents a significant challenge in polymorphic systems such as GQs, where precise control over environmental conditions is crucial. Here, we focus on the analysis of two well-characterized short sequences, Tel23 and c-Myc, selected as model systems of different conformations. Both these sequences have biological relevance. In particular, Tel23 (5′-TAGGGTTAGGGTTAGGGTTAGGG-3) belongs to telomeric sequences, typically short minisatellites arranged in tandem according to (TTAGGG)­n repeats, first detected at the chromosome ends in the majority of living organisms.^19^ The stabilization of these regions plays an important role in counteracting telomeric crisis, which is frequently observed in early stage epithelial neoplasms (e.g., colon, breast, cervix) prior to telomerase reactivation. Tel23 was recognized to adopt a three-layer hybrid-I GQ topology.^20,21^ The proto-oncogene c-Myc (5′-TGAGGGTGGGTAGGGTGGGGAA-3′), on the other hand, is among the most frequently implicated genes in human carcinogenesis—including Burkitt’s lymphoma as well as cervical, renal, and breast cancers,^22,23^ and assumes a parallel conformation in physiological buffers.^24^

## Materials and Methods

The oligonucleotide sequence Tel23 (5′-TAGGGTTAGGGTTAGGGTTAGGG-3) and c-Myc (5′-TGAGGGTGGGTAGGGTGGGGAA-3′) were purchased from Eurogentec (Belgium) and used without further purification. The lyophilized powders were dissolved in a 20 mM phosphate buffer at pH = 7, 0.3 mM EDTA, and 70 mM KCl. The solutions were heated to 95 °C for 5 min and then slowly cooled down to room temperature in ∼4 h. After this procedure, the samples were left at room temperature overnight. DNA concentration was determined from UV absorption measurements at 260 nm, using a molar extinction coefficient of 254600 M^–1^ cm^–1^ for c-Myc and 236500 M^–1^ cm^–1^ for Tel23. Samples for UVRR and CD measurements were prepared at 44 μM (Tel23) and 40 μM (c-Myc).

### CD Experiments

Circular dichroism experiments were done using Jasco J-810 spectropolarimeter on Tel23 and c-Myc, using a 1 mm path-length quartz cuvette. Spectra were recorded in the range from 220 to 330 nm, with a scan speed of 50 nm/min, by changing the temperature from 27 to 96 °C.

### UVRR Experiments

UVRR measurements were performed at the IUVS beamline at Elettra Sincrotrone Trieste using a synchrotron-based setup^25^ on the same samples measured through CD spectroscopy. A 10 mm path length quartz cuvette was used as sample holder. The excitation wavelength was set at 250 nm by regulating the undulator gap aperture and monochromatizing the incoming radiation through a 750 cm focal length spectrograph equipped with holographic gratings at 1800 and 3600 grooves/mm (Princeton Instruments). The Raman signal was collected in backscattering geometry and analyzed via a single-pass Czerny–Turner spectrometer (Princeton Instruments, 750 mm of focal length), equipped with holographic grating at 1800 g/mm, and detected using a UV-optimized CCD camera (resolution of about 2 cm^–1^/pixel). The calibration of the spectrometer was performed using cyclohexane (spectroscopic grade, Sigma-Aldrich). Beam power measured on the samples was about 4 μW.

## Results

The different topologies were identified through CD spectra (Figure 1a-b) collected from Tel23 and c-Myc solutions in the same K^+^ buffer, using characteristic markers for straightforward identification of their secondary structures. In most real cases, the rule applies that a positive CD at 260/295 nm and a negative CD at 240 nm indicate hybrid structures, while a positive CD at 260 nm and a negative CD at 240 nm are characteristic of parallel structures,^17,26–28^ as illustrated at the top of Figure 1, and in Supporting Information Figure S1.

**Figure 1:**
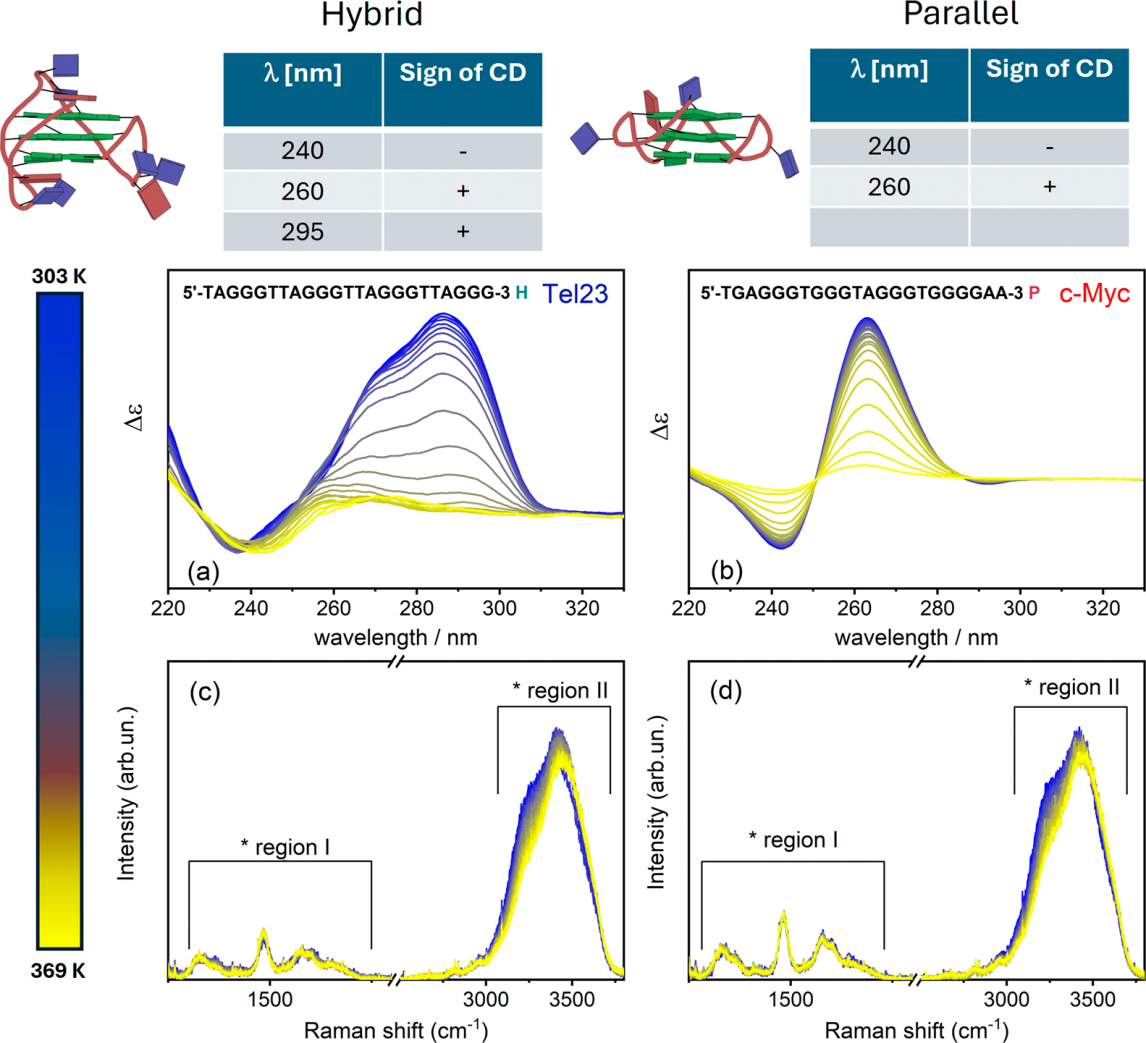
Tel23 and c-Myc CD profiles (a, b). Hybrid quadruplex DNA (Tel 23) shows positive bands around 295 and 260 nm and a negative band near 240 nm, while parallel DNA (c-Myc) exhibits a positive and a negative band at approximately 260 and 240 nm, respectively. (c, d) UVRR spectra, recorded with an exciting wavelength of 250 nm over the 1000–4000 cm^–1^ range, displaying two main spectral regions: region I, which is more sensitive to the solute, and region II, which is more influenced by the solvent. The structures of Tel23 and c-Myc on top were rendered from the PDB entries 2JSM^20^ and 1XAV,^24^ respectively.

In the same conditions, the vibrational features were detected by means of UVRR spectroscopy, using an excitation wavelength of 250 nm. The spectra, acquired over the 1000–4000 cm^–1^ wavenumber range (Figure 1c,d) at the IUVS beamline at Elettra Sincrotrone Trieste using a properly optimized synchrotron-based experimental setup,^29^ were processed as described in previous studies.^30,31^ The UVRR profiles reveal a GQ fingerprint region (1200–1800 cm^–1^) mainly due to solute vibrations (region I), along with a very intense band in the 3000–3900 cm^–1^ range, corresponding to the O–H stretching vibrational mode (region II), more closely associated with the intermolecular structure of the H-bond network of the solvent.^12^ In pure water and in very diluted aqueous solutions, one possible way to represent the broad band in region II is to decompose it into three components, centered at approximately ν_OH1_ ≈ 3200 cm^–1^, ν_OH2_ ≈ 3450 cm^–1^, and ν_OH3_ ≈ 3600 cm^–1^.^12,32,33^ The first component is mainly attributed to ice-like tetrahedral water structures, which exhibit strong intermolecular resonance coupling, with the OH oscillators phase-correlated with those of the nearest molecules.^34,35^ The central feature is generally related to associated water structures, where H-bonds are partially distorted, and phase correlation among vibrations of neighboring OH groups is lost.^36,37^ The peak at higher wavenumbers corresponds to OH groups that are only weakly stabilized by hydrogen bonding.^36,37^ It is worth noting that this decomposition approach is intrinsically linked to dynamic processes, in which distinct water species rearrange on the subpicosecond time scale. Vibrational spectroscopies, such as infrared and Raman, are powerful techniques for probing these short time scales, allowing discrimination among different H-bonded species and quantification of their relative area ratios. Given the low concentration of Tel23 and c-Myc in solution (∼40 μM), we applied this three-band decomposition to reconstruct region II of the UVRR spectra for both our solutions (see Figure S2). The fitting results, presented in the Supporting Information for the ordered and disordered hydrogen-bonding network contributions (see Figure S3), reveal some differences between the two systems investigated.

### Solute-Correlated Spectra

Based on this, the UVRR profiles of Tel23 and c-Myc were further processed to derive the solute-correlated (SC) spectra, capturing features from both solute intramolecular vibrations and solute-induced perturbations of the water OH stretching vibrations. Such perturbations are supposed to affect mainly vicinal water molecules, i.e. water molecules in close interaction with the solute, and thus are hereinafter referred to as *residual water* contributions. This widely adopted approach, originally proposed by Ben-Amotz et al.,^38,39^ is based on the idea that, in a highly dilute aqueous solution where solute–solute interactions are negligible, the observed spectrum can be described as a linear combination of three components: bulk water, solute molecules, and solute-induced perturbations of water molecules in the hydration shell. Under these conditions, the latter two contributions can be jointly referred to as solute-correlated (SC) spectra. Accordingly, the SC spectrum can be derived by determining the minimum-area, non-negative difference between the spectra of the solution and the pure bulk water.

The SC spectra shown in Figure 2a,b were obtained using the spectral differential method described above, by subtracting the pure buffer profile from that of the solution.^40^ Notably, with this approach, if the water structure in the hydration shell was identical to that of bulk water, the resulting SC spectrum would lack any residual OH spectral intensity.^41^ Region I, mainly associated with group vibrations of the solute subunits, is minimally affected by the subtraction (dashed areas in Figure 2a,b). On the other hand, region II shows a weak residual band after the bulk solvent contribution is removed. The residual intensity is small but outside the experimental errors.

**Figure 2:**
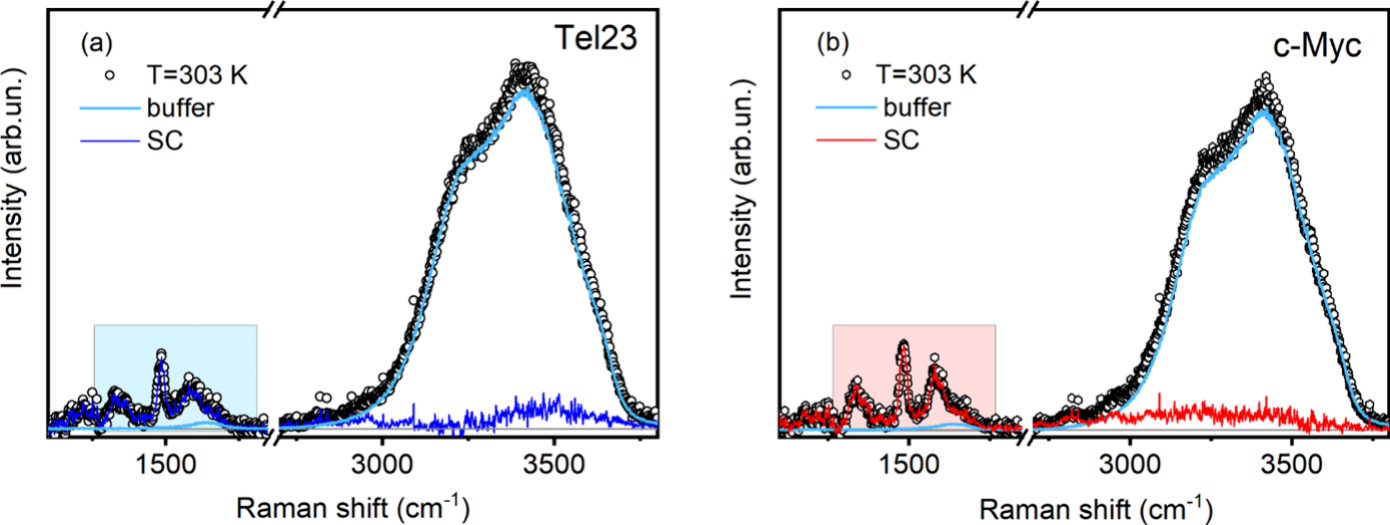
Tel23 (a) and c-Myc (b) UVRR solute-correlated difference spectra (SC). SC are achieved by subtracting the buffer profile from that of the solution to avoid negative contributions. The shaded areas highlight that, in region I, the differences between the UVRR solution spectra and the SC spectrum are minimal.

The determination of SC profiles effectively highlights the differences between Tel23 and c-Myc, both in the overall shape and temperature dependences. Comparing the low- and high-temperature SC spectra (Figure S4), we find that c-Myc shows less variation than Tel23, indicating greater thermal stability.

This difference likely arises from the intrinsic properties of the quadruplex topology and its interactions with water molecules, deserving further analysis of the two spectral regions. In region I, the contribution of the individual bases that constitute the GQ can be specifically enhanced depending on the excitation wavelength.^15^ It is known that at 250 nm, especially in the region between 1400 and 1700 cm^–1^, the UVRR signal is predominantly attributable to vibrations of dG residues, with minor contributions from dA and dT.^15^ This is verified here by reproducing the SC profiles as an additive combination of the UVRR spectra of the individual dG, dA, and dT signals detected at 250 nm (Figure S5), accounting for the correct number of bases present in each sequence.^42^ During the melting process, some bands may change their intensity or position, indicating a weakening of bonding strength and interaction among the involved molecular groups. Therefore, by fitting the data from both systems with the same ensemble of Gaussian functions (see Figure 3a,b), we were able to track the temperature behavior of the group vibrations mostly affected by the unfolding process.^14,30,42–44^ Owing to their distinct topologies, Tel23 and c-Myc exhibit different spectral changes as a function of temperature. To describe their thermal behavior, the following band indicators can be identified: G5, G8, G11, and G12 for Tel23 (Figure S6b–e) and G2 and G8 for c-Myc (Figure S7b,c). The G8 band (∼1484 cm^–1^), whose intensity varies in both systems, is one of the most characteristic Raman fingerprints of GQs. It is associated with the bending of C8–H as well as the stretching of N9–C8 and C8–N7 bonds in the dG moiety.^15^ G8 is particularly sensitive to guanine stacking: upon unfolding, the weakening of stacking induces a hyperchromic effect in absorption, consequently leading to an increase in the resonant Raman signal. We detected a greater increase in the intensity in Tel23, confirming its lower thermal stability compared to c-Myc. In Tel23, G5 (∼1375 cm^–1^) also changes with temperature. This contribution arises not only from dG vibrations but also from the exocyclic C5-CH_3_ deformation in dT residues, which are located in the external loops and, being exposed to solvent, are responsive to structural unfolding.^14^ Still in Tel23, the G11 (∼1580 cm^–1^) and G12 (∼1610 cm^–1^) bands, associated with interbase hydrogen bonding at the dG N2–H and N1–H positions, respectively, shift to lower frequencies upon GQ unfolding. This shift results from the replacement of interguanine hydrogen bonds with guanine-water interactions and occurs synchronously, as observed in the closely related Tel22.^30^ Although c-Myc is less temperature-dependent, we observe a progressive modification in the melting pathway of the G2 band (∼1318 cm^–1^), which is mainly associated with the dA ring C8–N9 and C2–N3 stretching modes.^45,46^ This modification is also partially influenced by the temperature variation of the peak at ∼1338 cm^–1^, which depends on the dG (C2′ *endo*/*anti*) guanosine conformations characteristic of parallel topologies.^45^

**Figure 3:**
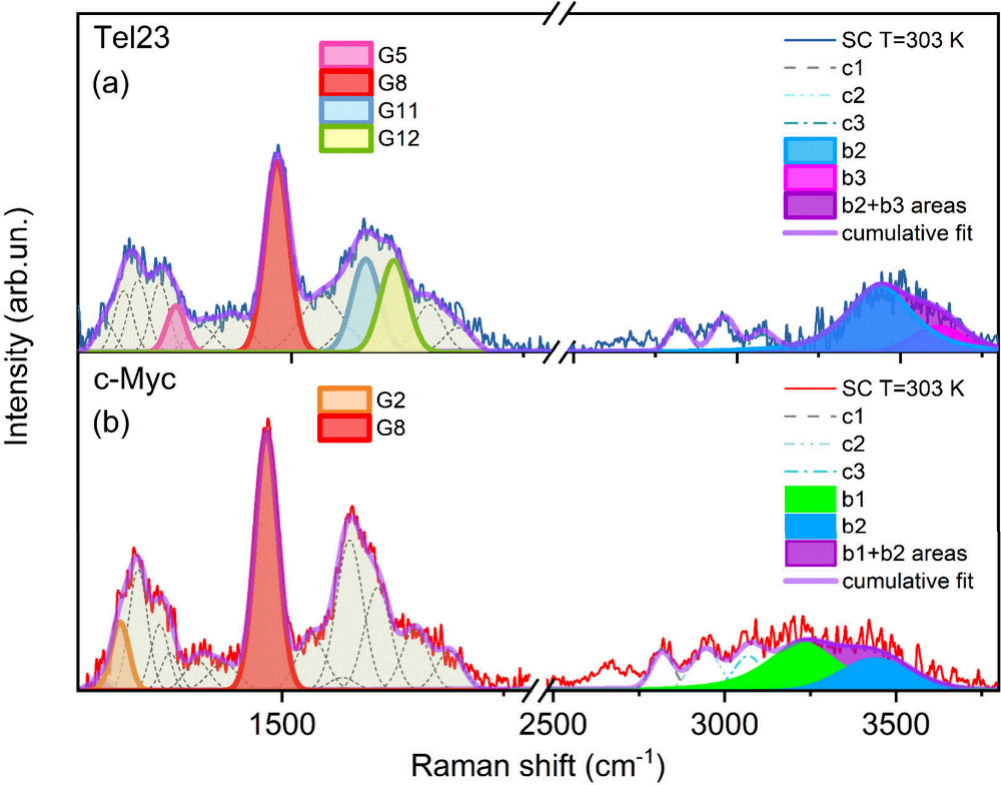
SC profiles of Tel23 (a) and c-Myc (b). Region I is modeled using a combination of Gaussian functions as described in the text. Tel23 and c-Myc respond differently to temperature. The most sensitive bands (G5, G8, G11, and G12 in Tel23 and G2 and G8 in c-Myc) are highlighted with colored areas. Region II can be reproduced by considering six peaks, labeled as c1, c2, c3, b1, b2, b3; c1, c2, and c3 are related to CH_3_ group vibrations, while b1, b2, and b3 bands are located approximately in the same position as the OH1, OH2, and OH3 bands shown in Figure S2.

Region II, rarely investigated in DNA solutions, emerges as the most intriguing part of the spectrum, where GQ-induced modifications to the solvent can be identified, enabling differentiation between the two sequences. The SC profiles show several features within the 2750–3850 cm^–1^ range, modeled using a minimal number of components, i.e. six Gaussian contributions c1, c2, c3, b1, b2, and b3.

The spectral region associated with the c1, c2, and c3 components falls outside the scope of this study; nonetheless, it is important to note that these features are common to both GQs and may be associated with CH stretching modes.^11,46^ Literature also refers to overtone bands linked to vibrational modes of guanine and adenine (though these are of lower stoichiometric relevance in GQs compared to guanine) detected within this energy range.^47,48^ Regarding the region above 3200 cm^–1^, which is the focus of this work, although minor contributions from overtones of lower-frequency GQ modes and from NH stretching cannot be entirely excluded, the positioning of bands b1, b2, and b3 near the characteristic spectral features of pure water supports their assignment to interacting water molecules. These bands can thus be primarily interpreted as OH stretching vibrations.

Our analysis reveals that for Tel23, a combination of the b2 and b3 bands is enough to reproduce the SC profiles, while for c-Myc, only the b1 and b2 bands are needed. The presence of these multiple contributions, which give rise to a broadened spectral shape, suggests distinct types of water molecules interacting with the solute. Interestingly, we observe that the total residual water in c-Myc (b1 + b2) is shifted to lower wavenumbers compared to that in Tel23 (b2 + b3), as clearly visible in Figure S8 where the first momentum, M_1_, which measures the central tendency of the residual water distribution, is calculated after removal of the CH contributions. In the case of c-Myc, *M*_1_ is 3324 cm^–1^, while in Tel23 it amounts to 3437 cm^–1^.^34^ This indicates that, in c-Myc, a portion of the residue is associated with a more structured network of interacting water molecules. This interpretation is consistent with studies on crystalline GQ structures, which emphasize that the nature of interacting water molecules is influenced by the geometry of loops and grooves. In particular, for some parallel GQ structures (i.e., PDBs 7KPL and 6N65), it has been hypothesized that, despite the absence of extended grooves, primary-sphere water molecules still cluster within them, particularly around the loops, thereby contributing to loop stabilization.^6^

Although these concepts have been established using atomistic techniques and their extension to our study remains speculative, the correspondence with our findings is highly interesting and lays the groundwork for an integrated multiscale approach, both computational and experimental. Furthermore, concentration-dependent investigations could provide deeper insights and allow for more comprehensive conclusions regarding the behavior of GQ solutions with different topologies.

## Discussion

The temperature trends of the (b2 + b3) region in Tel23 and of the (b1 + b2) region in c-Myc show a relative variation in Tel23 almost twice as large as in c-Myc (65% vs 35%), indicating that residual water in c-Myc persists even at high temperatures, as visible in Figure 4.

**Figure 4:**
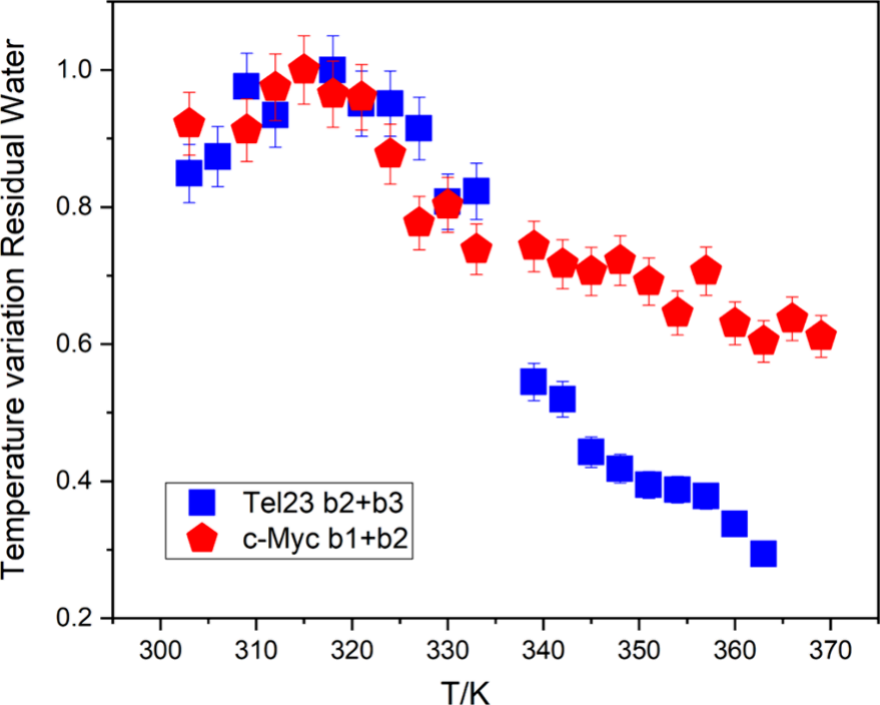
Temperature variation for the total residual water contribution for Tel23 (b2 + b3) and c-Myc (b1 + b2), normalized to the maximum value.

We also note that the individual bands of the two GQs, normalized to the total residual water contribution, namely, b2/(b2 + b3) for Tel23 and b2/(b1 + b2) for c-Myc, exhibit very peculiar temperature behaviors, with Tel23 showing a smoother trend compared to c-Myc. This implies that residual water participates in the structural modifications occurring during the melting process, which appears to be less cooperative (gradual) in Tel23 than in c-Myc (Figure 5a,b and Figure S9). The different degrees of cooperativity of folding/unfolding transitions of the two quadruplex topologies can be also monitored by CD, which track molar ellipticity at characteristic wavelengths, as reported in Figure S1. While for c-Myc, the trends at 240 and 260 nm evolve together, pointing to a relatively cooperative transition, the different behavior observed for Tel23 indicates the presence of intermediate states through which the system undergoes melting.^30^ It is particularly interesting to note the matching between the temperature dependence of b2/(b2 + b3) and b2/(b1 + b2), obtained from UVRR SC profiles of Tel23 and c-Myc respectively, and that of the corresponding CD ellipticity, measured at 260 nm (Figure 5a-b).

**Figure 5:**
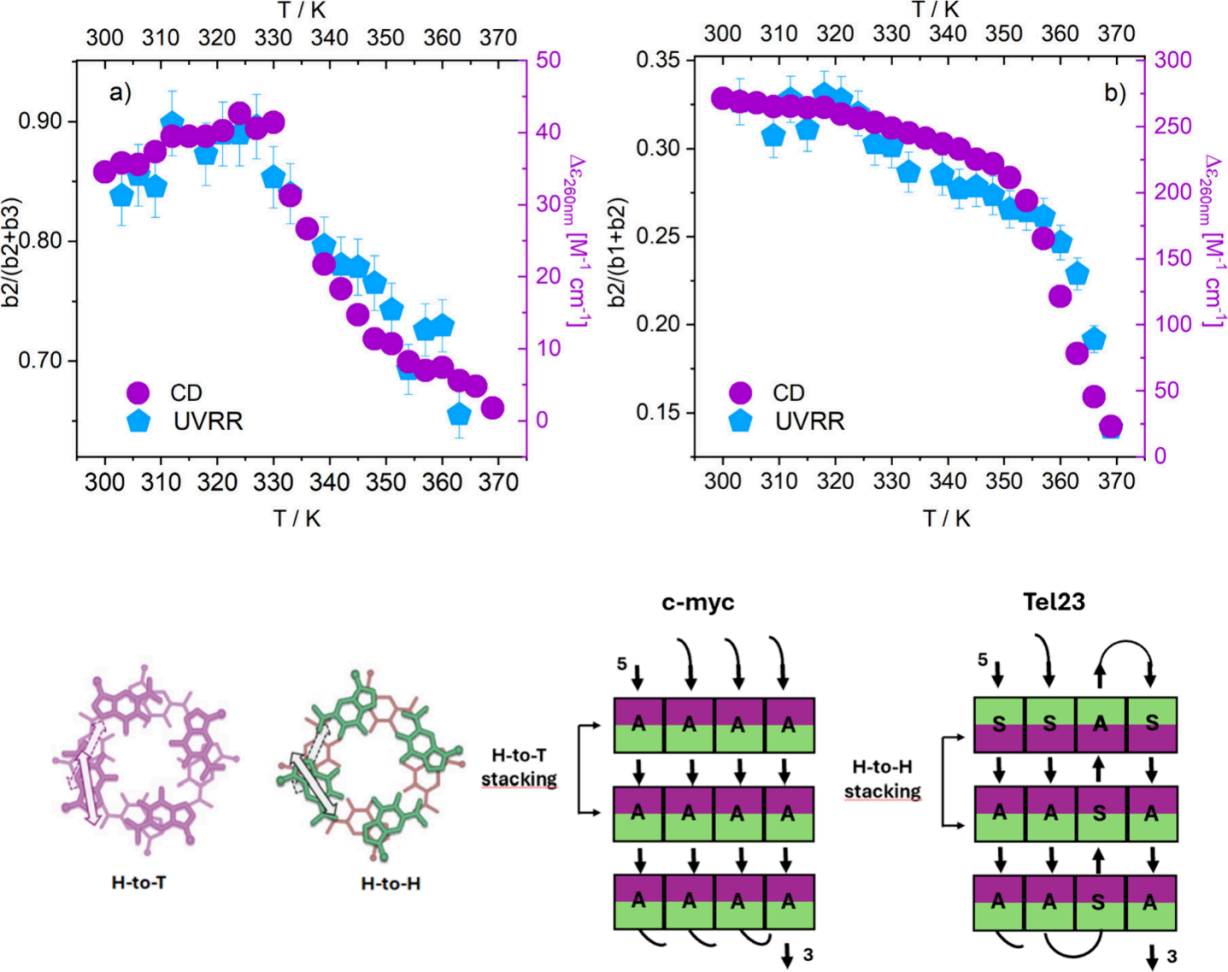
Top panel: Temperature dependence of the normalized b2 component (cyan symbols) and CD ellipticity taken at 260 nm (violet symbols) for Tel23 (a) and c-Myc (b). Bottom panel: Plan view of the stacking of two G-quartets: the “head” (H) and the “tail” (T) sides of the G-quartets are represented in purple and green, respectively (the double-head arrows represent the transition moments corresponding to the absorption band at ca. 250 nm), S and A refer to the *syn*- and *anti*-conformation around the glycosyl bond.^26^

The dominant features of GQs to CD signal in the UV–vis range have been primarily attributed to the electronic transitions of dG residues, with negligible contributions from other components of the biopolymer.^17,26,28,49^ Since stacked G-quartets can rotate relative to one another, they generate chiral exciton coupling between the transition dipole moments of neighboring guanines.^26,28^

The experimental CD spectra of main GQ topologies can then be qualitatively reproduced by considering only nearest-neighbor interactions between stacked G-quartets and accounting for their polarity dictated by the donor–acceptor direction of Hoogsteen hydrogen bonds that defines the quartet’s head and tail faces.^26,28,50^ Accordingly, a positive CD band near 260 nm turns out to be linked to the stacking of G-quartets with the same polarity, whereas a band at longer wavelengths (around 290 nm) arises from the stacking of quartets with alternating polarities.^26,28^

In our case, the parallel-stranded c-Myc features uniformly polarized G-quartets stacking head-to-tail (see scheme at bottom of Figure 5). Consequently, its CD spectrum shows a 260 nm maximum and a 240 nm minimum from positive exciton coupling.^28^ Tel23 adopts a 3 + 1 hybrid structure, with guanine sequences stacked in a mixed head-to-head (5′-middle) and head-to-tail (middle-3′) pattern between adjacent G-quartets. Its CD spectrum shows negative and positive bands near 240 and 260 nm, respectively, similar to c-Myc, along with an additional positive band around 290 nm.^26^ In particular, the 260 nm positive peak results from the overlap of contributions from the central tetrad interacting with both the 3′ (positive signal) and 5′ (negative signal) ends.^51,52^ It is likely the intrinsically mixed nature of this band that makes it a suitable marker for representing the global noncooperative melting process of Tel23, synchronized with the molecular vibrations of residual water. As a whole, the correlation reported in Figure 5, previously unexplored, suggests that, along the melting pathway, the distortion of hydrogen bond networks in the water near the solute is highly sensitive to the secondary structure of Tel23 and c-Myc.

## Conclusions

In conclusion, a detailed analysis of UVRR spectra allowed us to identify solute-induced perturbations of water OH stretch vibrations, defining specific indicators (b1, b2, and b3) for Tel23 and c-Myc quadruplex structures and characterizing their behavior during unfolding. Overall, the temperature-dependent variation of total residual water is smaller in c-Myc than in Tel23. In the former, solute-correlated water appears at lower wavenumbers, suggesting a more ordered OH network that may underlie its high thermal stability. Furthermore, tracking individual residual water components, normalized to the total, reveals that they qualitatively mirror key features of the melting process, as monitored through secondary structure variations.

We thus demonstrate that coupling UVRR and CD spectroscopies enables the mapping of distinct regions within the GQ conformational landscape at micromolar concentrations, providing a powerful approach to correlate structural and vibrational properties even in spectral regions dominated by solvent contributions. To generalize these findings, the investigation should encompass various GQ sequences, including antiparallel ones. As a final and promising insight, it is worth underlining that the subtraction method used to calculate residual water in our UVRR spectra was recently applied to different cell lines, to estimate the population of biointerfacial water located in the vicinity of biomolecules.^53^ This paves the way for characterizing structural water in G-quadruplexes, even in crowded environments or in cells.

## Supplementary Material



## Notes

Additional experimental details including CD spectra temperature behavior (Figure S1), O–H stretching band (Figures S2 and S3), difference solute-correlated (SC) spectral profiles (Figure S4), SC profiles reconstruction (Figure S5), GQ fingerprint regions (Figures S6 and S7), first momentum of the residual water contribution (Figure S8), and normalized behavior of the residual water components (Figure S9) (PDF)
