# Supplementary material for: Probing Residual Water in G‑Quadruplex Structures through Molecular Vibrations
Source: J Phys Chem B. 2025 Sep 19;129(39):9965–72. doi: 10.1021/acs.jpcb.5c04949 (PMC12498502; doi:10.1021/acs.jpcb.5c04949)
Supplement: Supplementary file 1 [file jp5c04949_si_001.pdf]

Supporting Material

Probing Residual Water in G-Quadruplex Structures through Molecular Vibrations

Valeria Libera,[1] Sara Catalini,[2,3] Francesca Ripanti,[4] Luca Bertini,[1] Martina Alunni Cardinali,[5] Francesco D'Amico,[6] Andrea Orecchini,[1,7] Caterina Petrillo,[1] Marco Paolantoni,[5] Alessandro Paciaroni,[1,*] Lucia Comez[7,*]

[1]Dipartimento di Fisica e Geologia, Università di Perugia, 06123 Via Pascoli, Perugia, Italy

[2]Department of Chemistry, University of Basel, St. Johanns-Ring 19, CH-4056 Basel, Switzerland

[3]European Laboratory for Non-Linear Spectroscopy, Via Nello Carrara 1, 50019 Sesto Fiorentino, FI, Italy

[4]Department of Life and Environmental Sciences, Polytechnic University of Marche, Via Brecce Bianche, Ancona, 60131, Italy

[5]Dipartimento di Chimica, Biologia e Biotecnologie, Università di Perugia, Via Elce di Sotto 8 - 06123 Perugia, Italy

[6]Elettra-Sincrotrone Trieste, Strada Statale 14 - km 163,5 in AREA Science Park 34149 Basovizza, Trieste, Italy

[7]Istituto Officina dei Materiali (IOM) - CNR 06123 Via Pascoli, Perugia, Italy

Corresponding Authors: *alessandro.paciaroni@unipg.it, comez@iom.cnr.it

TABLE of CONTENTS

CIRCULAR DICHROISM

Figure S1 *Temperature behavior of Tel23 and c-Myc CD spectra*

UVRR SPECTRA

Figure S2 *O-H stretching band*

Figure S3 *OH1 and OH2 components as a function of temperature*

Figure S4 *Difference solute-correlated (SC) spectral profiles*

Figure S5 *Reconstruction of the SC profiles in region I in terms of A, T, and G bases*

Figure S6 *GQ fingerprint region (Tel23)*

Figure S7 *GQ fingerprint region (c-Myc)*

Figure S8 *First momentum of the Residual Water contribution*

Figure S9 *Normalized behavior of the Residual Water components*

CIRCULAR DICHROISM

Temperature behavior of Tel23 and c-Myc CD spectra

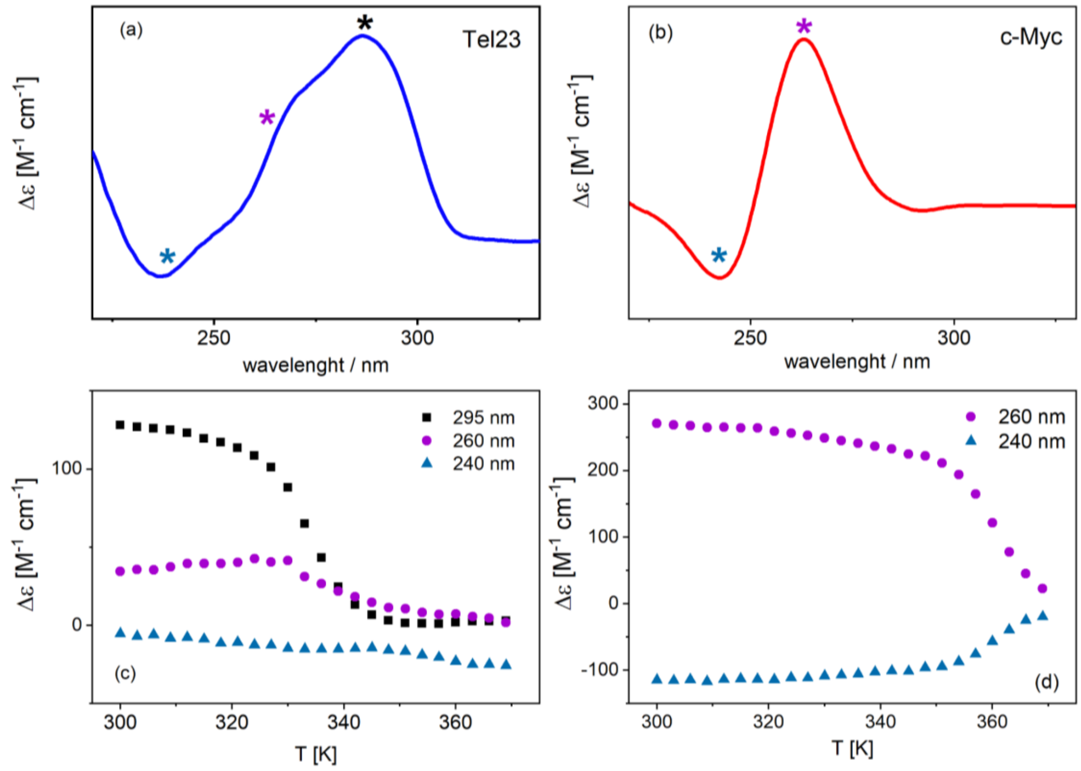

Figure S1 Tel23 and c-Myc CD spectra at ambient temperature (a-b). Temperature behavior of ellipticity at specific markers for hybrid (240, 260, 295 nm) and parallel (240, 260 nm) conformers (c-d). The unfolding pathway of Tel23 presents intermediate states, indicated by the different behavior at the three wavelengths (c); by contrast, for c-Myc, the trends at 240 and 260 nm exhibit inflection points at the same temperature, suggesting an almost two-state transition (d).

UVRR SPECTRA

O-H stretching band

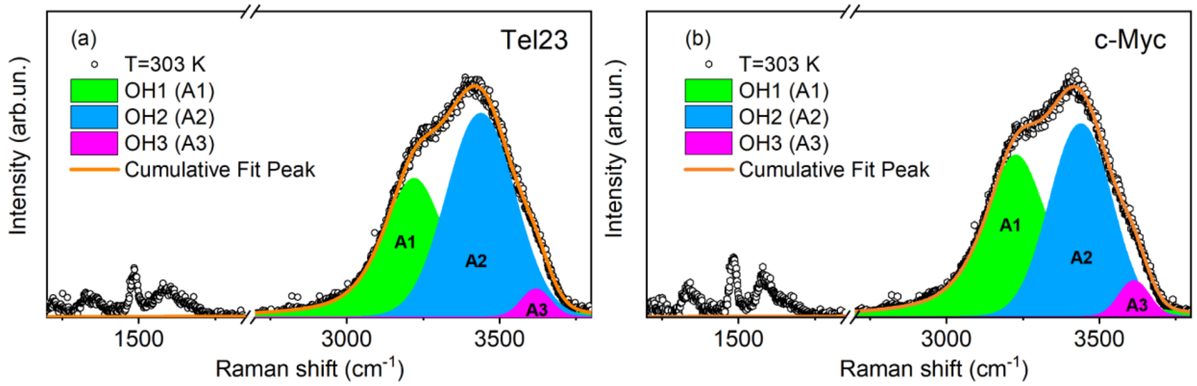

Figure S2 UVRR profiles of Tel23 (a) and c-Myc (b) aqueous solutions. A multicomponent fitting procedure (colored peak areas) performed over region II, yields a cumulative curve (orange lines) that provides an excellent fit to the experimental data. The single components, namely, A1, A2, and A3, are associated to different OH contributions, centered at approximately $\nu_{OH1} \approx 3200$ cm^{-1}, $\nu_{OH2} \approx 3450$ cm^{-1}, and $\nu_{OH3} \approx 3600$ cm^{-1}, whose nature is described in the main text.

OH1 and OH2 components as a function of temperature

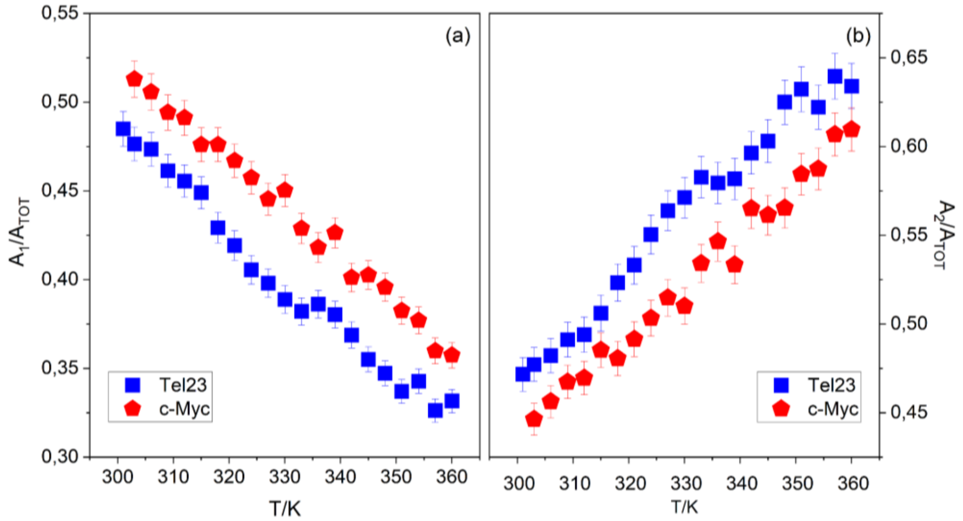

Figure S3 The relevant A_1 (a) and A_2 (b) areas normalized over the total contribution ($A_{TOT}=A_1+A_2+A_3$) are reported for Tel23 and c-Myc.

Difference solute-correlated (SC) spectral profiles

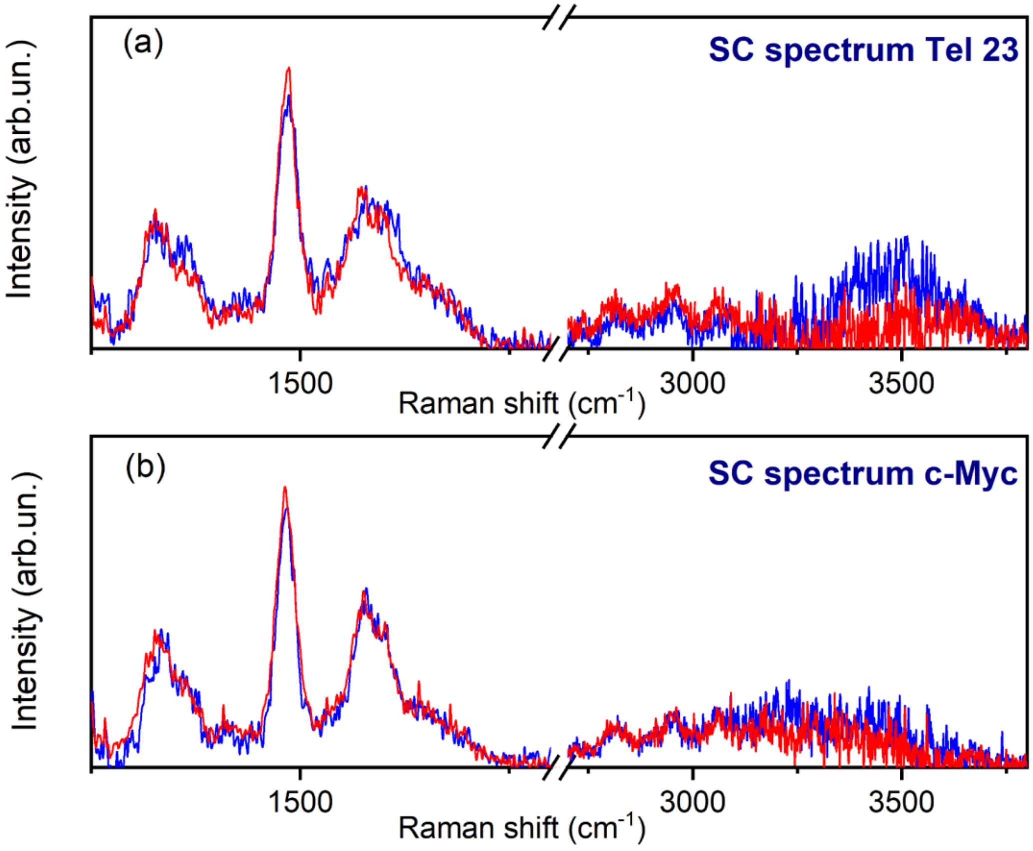

Figure S4 Tel23 (a) and c-Myc (b) UVRR solute-correlated spectra (SC) at ambient (blue) and high (red) temperature.

Reconstruction of the SC profiles in region I in terms of A, T, and G bases

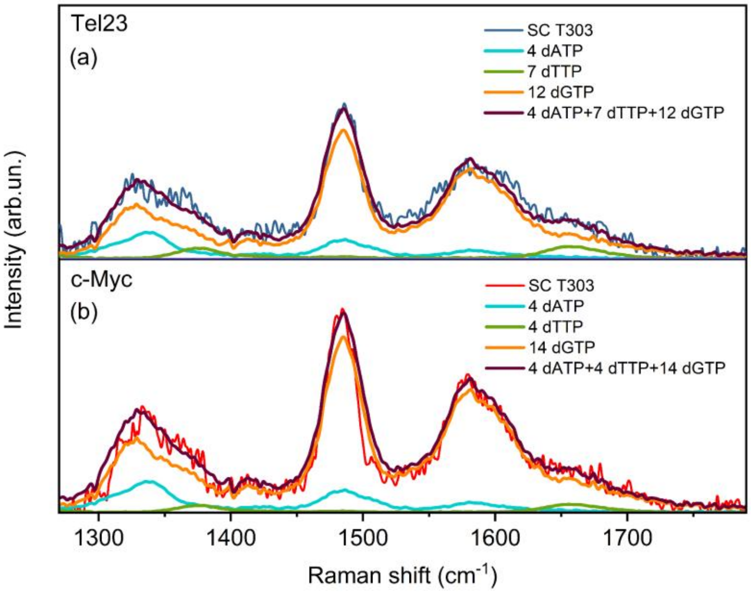

Figure S5 SC profiles of Tel23 (a) and c-Myc (b). Region I is modeled using a combination of the UVRR spectral profiles of dATP, dTTP, and dGTP collected with a 250 nm excitation wavelength, [1] taking into account the appropriate number of adenines, thymines, and guanines depending on the quadruplex sequence.

GQ fingerprint region

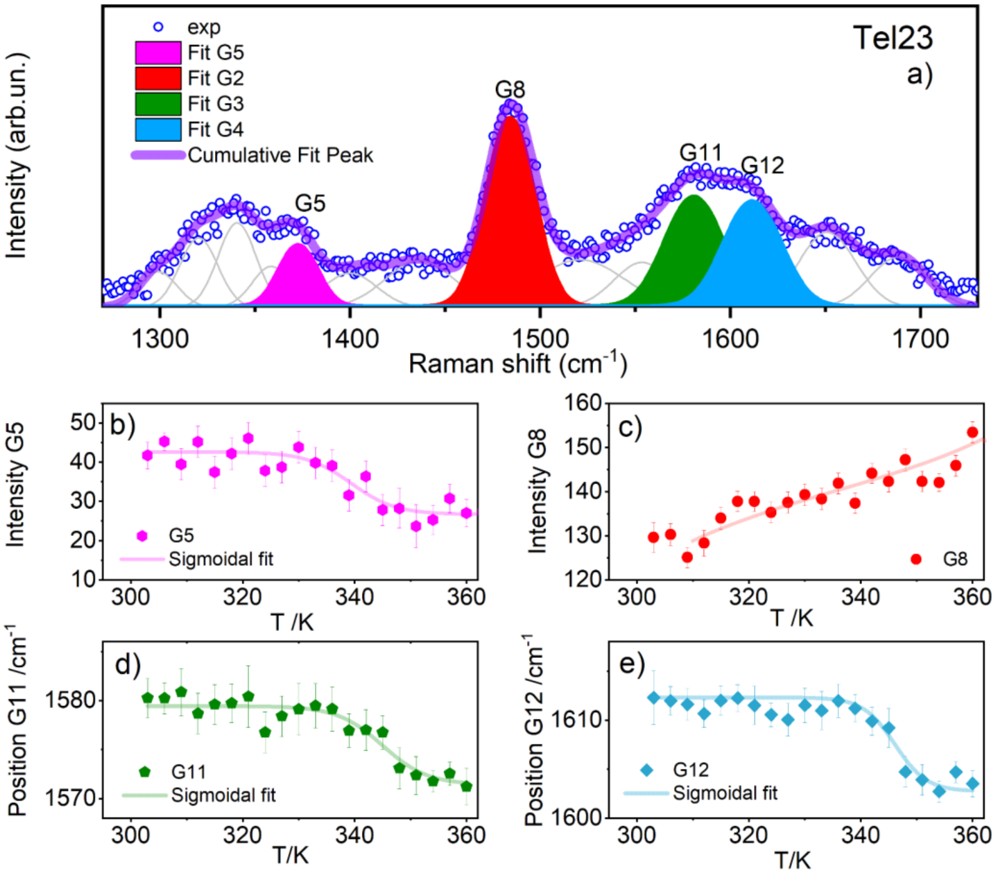

Figure S6 Representative UVRR spectra for Tel23 (a), reported together with the fit curves of the individual Gaussian components and the cumulative fit (given by the sum of the 14 Gaussian functions).[2] The colored areas refer to the bands that show distinct temperature trends. Bottom panels (b-c) refer to the temperature behavior of the intensity of G5 and G8 bands, while panels (d-e) refer to the frequency shift of the G11 and G12 bands.

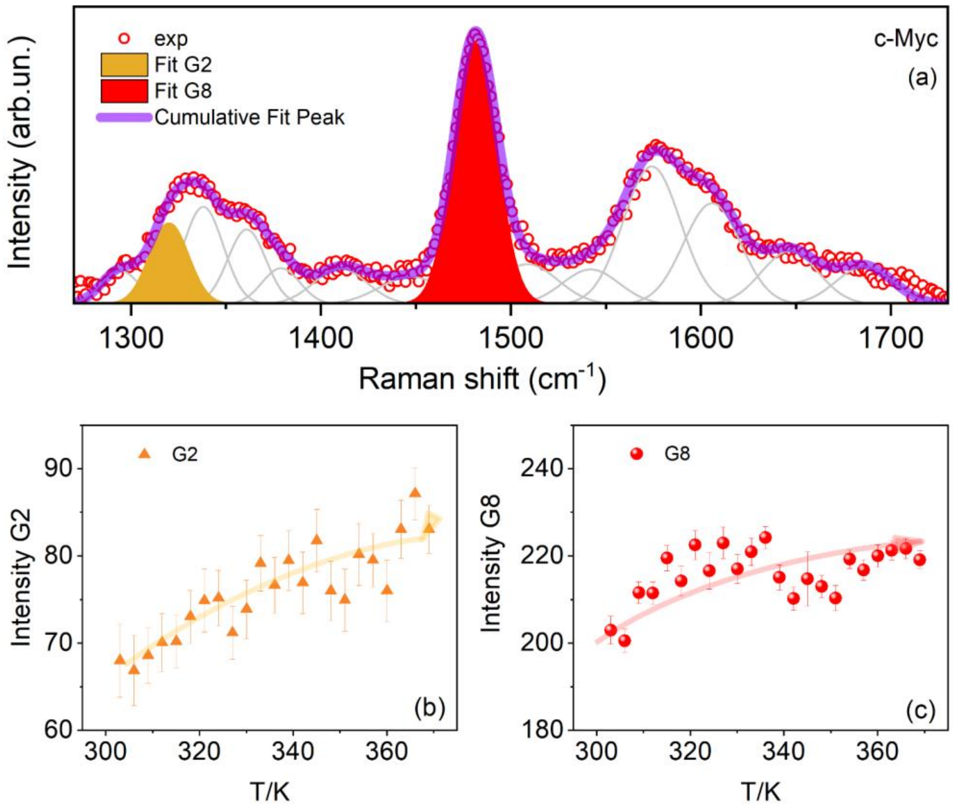

Figure S7 Representative UVRR spectra for c-Myc (a), reported together with the fit curves of the individual Gaussian components and the cumulative fit (given by the sum of the 14 Gaussian functions).[2] The colored areas refer to the bands that show distinct temperature trends. Bottom panels (b-c) refer to the temperature behavior of the intensity of G2 and G8 bands.

First momentum of the Residual Water contribution

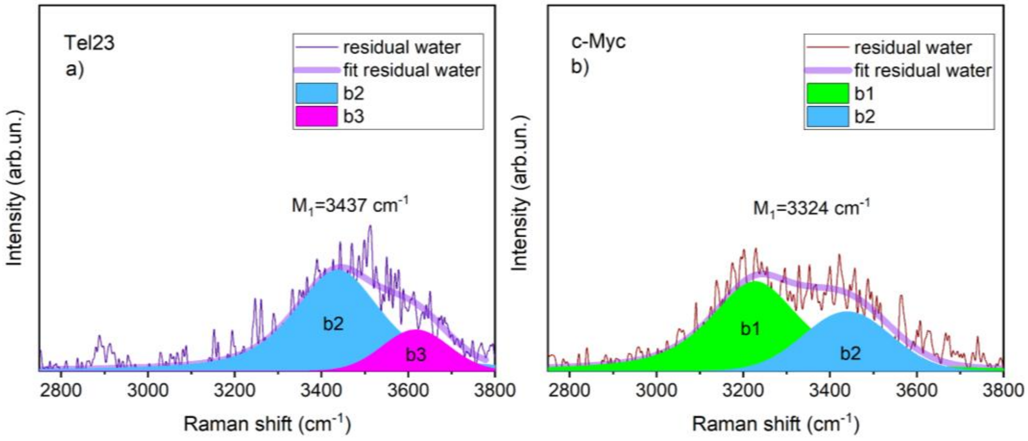

Figure S8 Tel23 (a) and c-Myc (b) UVRR smoothed SC spectra at 303 K. The OH stretching region, attributed to residual water, is shown after the removal of CH contributions, which were modeled using three Gaussian profiles, as described in the text and shown in Figure 3.

Normalized behavior of the Residual Water components

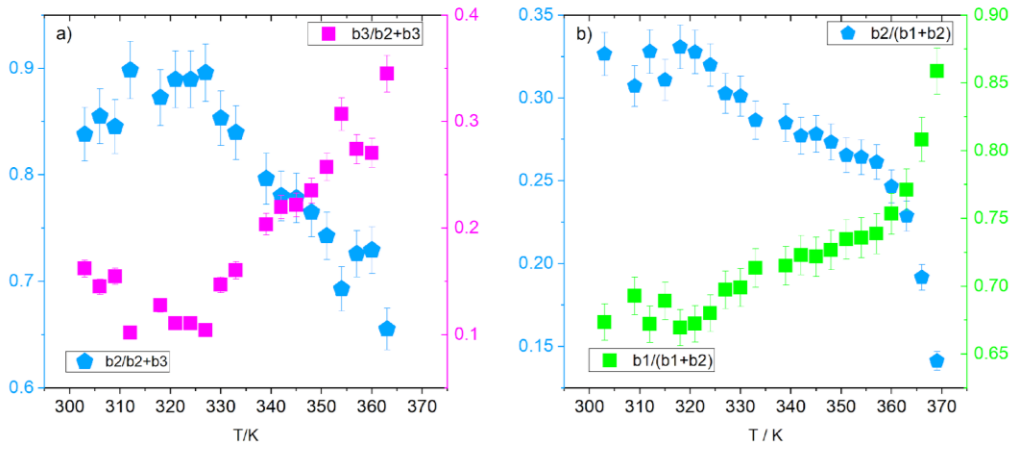

Figure S9 Temperature variation for the normalized residual water contributions, namely b2/(b2+b3) and b3/(b2+b3) for Tel23 (a) and b1/(b1+b2) and b2/(b1+b2) for c-Myc (b).

REFERENCES

(1) D'Amico, F.; Zucchiatti, P.; Latella, K.; Pachetti, M.; Gessini, A.; Masciovecchio, C.; Vaccari, L.; Pascolo, L. Investigation of Genomic DNA Methylation by Ultraviolet Resonant Raman Spectroscopy. *J Biophotonics* **2020**, *13* (12), e202000150.

(2) Libera, V.; Bianchi, F.; Rossi, B.; D'Amico, F.; Masciovecchio, C.; Petrillo, C.; Sacchetti, F.; Paciaroni, A.; Comez, L. Solvent Vibrations as a Proxy of the Telomere G-Quadruplex Rearrangements across Thermal Unfolding. *Int J Mol Sci* **2022**, *23* (9). https://doi.org/10.3390/ijms23095123.
